# Sexual Influence of Anæsthetics

**Published:** 1874-11

**Authors:** 


*THE CLINIC—September*, 1874.

INFLUENCE OF ANÆSEHETICS UPON THE SEXUAL IMPRESSIONS OF FEMALES.—A physician, called as an expert before a United States tribunal, made the following declaration : "A woman under the influence of anæsthesia is more liable to conception than when sexual intercourse has happened by force, and I concur in the opinion of Dr. Beck, expressed in his treatise on medical jurisprudence, that women may conceive during anæsthesia. The relaxation it produces facilitates conception."

This point seems to me established ; but I desire to add an observation which I have made in my practice, and one that it deeply concerns physicians to know. It is well known to-day that occasionally, under the influence of ether or chloroform, an excitation of the sexual organs is produced, and a feeling is excited in the mind by this sensation which may make a woman believe that she has been subjected to violence.

The first case of this nature which I witnessed myself occurred during a delivery. The woman, placed under chloroform, experienced sensual sensations so vivid that she accused me of having violated her, and called on her husband for protection. But he had been with her all the time, as well as a dozen women who had never quitted the chamber.

In a second case, I was administering chloroform to a woman to have a tooth extracted, but the physiognomy of the patient showed an expression of veneral excitement so pronounced that I hastened to call in her parents. On awaking, she seemed astonished to see herself surrounded by her family, and clearly exhibited what her impressions had been.

On another occasion, a lady of a certain age entered my office, in a state of high excitement, and related that she had gone to her surgeon to have a trivial operation performed, to relieve the pain of which she had taken chloroform, and the surgeon had abused her while under its influence. I was persuaded that she had deceived herself, and on examining all the circumstances, perfectly proved to her that she had been subject to a delusion.

The moral is, that physicians should never administer ether or chloroform except in the presence of witnesses.—*Revue Medicale, August*, 1874.

